# Editor's Letter-Box

**Published:** 1902-06-14

**Authors:** 


### Editor's Letter-Box.

[Our Correspondents are reminded that prolixity is a great bar to publication, and that brevity of style and conciseness of statement greatly facilitate early insertion.]

"T. C. E." writes: Your interesting article on "Punctuality in Infant-Feeding" recalled to my mind a question on which I have heard divergent opinions, viz., is the morning or the afternoon milk of cows the better? Two or three housekeepers have assured me that morning milk is better, and make it their practice to try for all the morning milk that can be spared them. On the other hand, I fancy I have seen the converse stated in your columns; and having regard to the statements made in your recent article, it would seem that my housekeeper friends are probably wrong, there being a comparatively short time between morning and afternoon milking. It may be, however, that while a 16 hours' wait deteriorates the milk, a 12 hours' wait does not have that effect. It would be very instructive if you could give any information on this point. I should much like to know also in what way the milk deteriorates—whether it becomes at all harmful, or merely poorer in quality. If the former, mothers who can only suckle their children at night might find it well to draw off the first supply.

[The evidence on which we commented was to the effect that by unduly lengthening the interval between the times of milking the quality of the milk was deteriorated. If one is asked to explain why this should be the story becomes a long one. We may say, however, that the formation of milk is not a process which takes place, as one may describe it, "of itself," nor is it a process which goes on equally all the time. It depends much upon the stimulus applied to the teat or nipple, and also upon that supplied by the complete emptying of the tubes, and it would seem that any undue lengthening of the interval between the milkings may disturb the normal in this regard. As to the comparative advantages of morning and evening milk, this must be remembered, that all the manipulations, milking, canning, etc., of the morning milk are performed before the heat of the day has come on, and that in summer time the opposite is the case with the milk drawn in the afternoon. Of course the morning milk in large towns is often that drawn in the afternoon in some distant country place.—Ed. The Hospital.]

